# A simulation-based module in pharmacology education reveals and addresses medical students’ deficits in leading prescription talks

**DOI:** 10.1007/s00210-021-02151-w

**Published:** 2021-09-15

**Authors:** Verena Kirsch, Jan Matthes

**Affiliations:** Center of Pharmacology, Institute II, University of Cologne, Gleueler Strasse 24, 50931 Cologne, Germany

**Keywords:** Health communication, Drug prescribing, Drug information, Guide, Medical education

## Abstract

Although doctor-patient communication is essential for drug prescription, the literature reveals deficits in this area. An educational approach at the Cologne medical faculty aims at identifying and addressing those deficits in medical students.

Fifth-year medical students first conducted a simulated prescription talk spontaneously. Subsequently, the conversation was discussed with peer students. A pharmacist moderated the discussion based upon a previously developed conversation guide. Afterwards, the same student had the conversation again, but as if for the first time. Conversations were video-recorded, transcribed and subjected to quantitative content analysis. Four days after the simulation, the students who conducted the talk, those who observed and discussed it, and students who did neither, completed a written test that focused on the content of an effective prescription talk.

Content analysis revealed clear deficits in spontaneously led prescription talks. Even essential information as on adverse drug reactions were often lacking. Prescription talks became clearly more informative and comprehensive after the short, guided peer discussion. With regard to a comprehensive, informative prescription talk, the written test showed that both the students who conducted the talk and those who only observed it performed clearly better than the students who did not participate in the educational approach.

Deficits regarding prescription talks are present in 5^th^ year medical students. We provide an approach to both identify and address these deficits. It thus may be an example for training medical students in simulated and clinical environments like the EACPT recommended to improve pharmacology education.

**Supplementary Information:**

The online version contains supplementary material available at 10.1007/s00210-021-02151-w.

## Introduction

Prescribing is a key element of the medication process and can therefore contribute to a safe drug therapy (Möller and Aly 2012). Medication is prescribed frequently, about during every second physician–patient consultation (Stevenson et al. 2000; Richard and Lussier 2006). Patients consider physicians to be their primary source of medical information (Tarn et al. 2009b). However, they also feel the need for improvement, mainly regarding information about potential risks and adverse drug events (Barry et al. 2000; Ziegler et al. 2001). Especially in these fields, information is often missing in medical communication. Studies show that risks and adverse drug events are only addressed in about one third of all prescription talks (Makoul et al. 1995; Tarn et al. 2006). This poor communication enhances the risk for medication errors such as non-adherence (Osterberg and Blaschke 2005) or dissatisfaction regarding a medication therapy (Stevenson et al. 2000; Richard and Lussier 2006, 2007). Non-adherence is associated with a reduction in treatment efficacy while an improved adherence on the other hand can contribute to saving costs and improving patient-relevant outcomes (Sokol et al. 2005; Simpson et al. 2006; Kripalani et al. 2007; Matthes and Albus 2014). For safe prescription, it is also important to involve patients in the medical process. Not only do patients like to be involved, their participation also correlates with the ability to recall the given information (Dillon 2012; Altin and Stock 2016; Milky and Thomas 2020). In practice, however, decisions are often made by the physicians alone (Loh et al. 2007; Karnieli-Miller and Eisikovits 2009). One reason for physicians’ deficits in medical communication is the neglect of the topic in medical education (Richir et al. 2008; Rothwell et al. 2012). New prescribers themselves have pointed to deficits in both undergraduate and postgraduate education in prescribing (Tobaiqy et al. 2007; Heaton et al. 2008). The foundation for prescribing behaviour is already laid in medical school. One cause of prescribing errors seems to be deficits in applying knowledge in a realistic setting (Aronson 2006; Singh and Pushkin 2019). In a previous study, we asked 816 medical students at different stages to self-assess their deficits in pharmacology (Johannsen et al. 2019). Throughout, they assessed their application-oriented knowledge lower than their declarative knowledge. In prescription talks with simulated inpatients, 5^th^ year medical students showed great deficits in medication communication skills regarding drug prescriptions (Hauser and Matthes 2017). Essential information was often lacking, e.g. only a few students addressed potential adverse effects. These findings demonstrate the need for (more) application-oriented training of medical students including medication communication.

Here we describe a teaching approach aimed at effective doctor-patient communication during a prescription talk. The analysis of conversations with simulated outpatients reveals students’ actual deficits and thus shows their specific training needs. The learning gain of the students regarding a prescription talk, which is provided by our simple approach, is evident both immediately and even a few days later.

## Material and methods

### Setting and participating students

Medical studies in Germany take at least 6 years (12 terms) and include three sections: 2 years of preclinical studies followed by 3 years of clinical studies and a final practical year. Regarding the pharmacology education in Cologne there are two mandatory main courses, taking place in the 6^th^ and 9^th^ term. Medical communication aiming at drug prescription so far was only focused by an elective (Hauser et al. 2017) and in parts during the “PJ-STArT-Block” (Kirsch et al. 2019), a one-week course preparing medical students for their final practical year (see below).

The majority of the students participating in the pharmacology module described here had completed both main pharmacology courses (basic and clinical pharmacology) successfully. During the study period of four terms (summer 2018 to winter 2019), 224 students attended the module. A total of 56 students conducted a simulated prescription talk by him- or herself (as “doctors”), the others observed and discussed the simulated medical communication (see “**The pharmacology module on prescription talks****”** and results section). 38 students who played the doctor agreed to have their conversation simulation filmed and analysed as part of this study. In addition, 61 students attending the one-week course but not the pharmacology module described here volunteered in a written test all participants were asked to take (“**The pharmacology module on prescription talks****” section**).

### The pharmacology module on prescription talks

The pharmacology module described here was developed as a part of the one-week course “PJ-STArT-Block” preparing 5^th^ year medical students for their final practical year (STArT: Schlüsselkompetenz-Training und Anwendung in realitätsnahen Tagesabläufen; in English: key-competence training and application in realistic daily routines). Within this course a total of four pharmacology modules aim at an effective and safe drug treatment with a focus set on doctor-patient communication (Kirsch et al. 2019). The module introduced here is described in detail in the results section. In brief, one student plays the doctor and is to lead a conversation with a simulation patient (i.e. an actor or actress). In this consultation, an antibiotic drug treatment is to be prescribed for a community-acquired pneumonia on the background of the patient’s chronic obstructive pulmonary disease (COPD) and heart failure. In a first run, the talk is spontaneous and based upon only these essential information (“impromptu talk”). Immediately afterwards, a pharmacist moderates a short discussion of the student in charge with three fellow students who observed the conversation. This discussion is based upon a recently developed conversation guide (“**The conversation guide****” section**) handed over to the students after the impromptu talk. The same student is then to give it a second try, i.e. to lead the prescription talk again, as if it were the first time. Both conversations were filmed to allow for content analysis. The role of the patient is played by professional actresses or actors. To further standardise the setting, all actors or actresses had the same role script and were told to just answer questions and not to ask about relevant information themselves.

During the last two terms (summer and winter 2019), all students attending the one-week “PJ-STArT-Block” were asked to volunteer a written test four days after the pharmacology module. The case-based task was to describe a prescription talk with the case vignette describing a patient suffering from non-complicated bacterial cystitis. The content of the answers was then subjected to an exploratory analysis with respect to the aspects considered in the simulated prescription talks. 14 students who played the doctor, 26 who watched and discussed this scenario, and 61 students attending the one-week course but not the pharmacology module described here took the test.

### The conversation guide

The guide used in our approach was previously developed as an aid in conversations about an upcoming drug prescription (Hauser and Matthes 2017). Together with medical students, the guide was discussed, adjusted and then tested in simulated prescription talks before. In these simulations, the guide proved to be reliable and discriminative when used as a checklist (Hauser et al. 2017). The guide contains informative aspects of an effective and safe drug therapy, as well as the basic steps of shared decision-making (Loh et al. 2007; Tarn et al. 2009a; Elwyn et al. 2012). Regarding the patient-relevant drug information, the guide is based on the Medication Communication Index (MCI), including the purpose of treatment, duration of treatment, instructions for use (e.g., quantity and dosage), as well as risks and adverse drug effects and their probabilities. Recently, a study indicated this conversation guide to improve doctor-patient communication in GP practices (Kirsch and Matthes 2021).

### Content analysis

Students conducted the simulated prescription talk twice, first without knowing the above-mentioned conversation guide and then after a peer discussion considering the guide. The prescription talks were videotaped and transcribed based on a predefined protocol using the software MAXQDA version 2018 for qualitative data analysis (VERBI Company Ltd. Berlin, Germany). We applied a content analysis according to Mayring on the anonymised transcripts (Mayring 2010). Categories were formulated deductively considering the conversation guide mentioned above (Hauser and Matthes 2017; Kirsch and Matthes 2021). Assignment of conversation passages to categories was based upon identified anchor examples and coding rules for each deductive category. Sub-categories were formed depending on the breadth in which a category showed up in the conversations. Within a feedback loop, the two authors revised the categories within the process of analysing independently and checked them in respect to their reliability. Whenever interpretations of the code were divergent, a consensus was reached by discussing that particular text passage. As suggested by Mayring, we subjected the identified codes to an exploratory frequency analysis (‘quantitative content analysis’) to compare the prescription talks both with each other and each before and after the intervention (Mayring 2010). Regardless of how many subcategories were addressed or how often a main category was touched during a talk, it was counted only once.

### Statistical analysis and ethical issues

Using contingency tables, we compared the frequency of mentioning aspects in the simulated prescription talks and in the voluntary written test, respectively. A p-value < 0.05 in Fisher’s exact test was considered indicating a statistically significant difference. The described pharmacology module was implemented as part of the regular medical studies and not for scientific reasons. It could neither affect students’ study progress nor grades. Our analyses are furthermore authorised by a student consent form signed when enrolling to medical studies at the University of Cologne (‘Declaration of voluntariness of attending the reformed medical curriculum’, including an agreement regarding “the collection, storage and scientific evaluation of my personal data” and “data concerning my studies (e.g. study duration, exam results)”). The local Ethics Committee raised no concerns (ref. 21–1425-retro).

## Results

### The impromptu prescription talk

#### Procedure of the first encounter

The first encounter was analysed in order to collect a status quo regarding the medical communication skills of the students and used in terms of a need analysis. One out of a group of four students acts as a doctor and is supposed to have a prescription talk for an antibiotic drug treatment with a patient simulated by an actor or actress. Besides the diagnoses heart failure (treated for three years), COPD (treated for six months) and community acquired pneumonia (CAP) as diagnosed by just made physical examination and chest x-ray, no further information is given about the patient. The “doctor” should agree with his or her fellows very briefly and without extensive discussion on the initial drug treatment of CAP. He or she then immediately enters the doctor’s office where the patient is waiting, while the fellow students and the tutor observe the impromptu conversation through a mirrored window. The “doctor” ends the prescription talk independently when he or she feels that all important information has been gathered or given.

#### Students’ medical communication in the impromptu prescription talk

Some contents were already mentioned frequently in the spontaneous conversation, others rather rarely (Table 1). For example, all students gave information about the purpose of treatment and the name of the new medication. In contrast, only two of 38 addressed possible adverse drug effects. Most students (32 of 38) touched the aspect of instructions for use in some way, at least by using the term “tablet”. Only 14 out of 38 students obtained consent from the patient. In short, impromptu prescription talks revealed clear deficits with regard to the medical aspects addressed.

**Table 1:** Frequency of aspects mentioned in an impromptu (1^st^ encounter) simulated prescription talk or the simulated prescription talk led by the same student after a short, guided peer discussion (2^nd^ encounter), respectively

Main category	Frequency during 1^st^ encounters (n = 38 in total)	Frequency during 2^nd^ encounters (n = 38 in total)
Adverse drug effects	2	37
Mechanism of action	4	8
Prognosis	9	32
Consent	14	29
Progress evaluation	15	31
Allergies	24	36
Drug history	25	37
Setting	30	25
Instruction for use	32	38
Pre-existing diseases	34	35
Purpose of treatment	38	38
Naming new medication	38	38

### Second try after guided peer discussion

#### Peer discussion between first encounter and second try

After returning from the patient, the “doctor” immediately discusses the situation with his or her fellow students. Subjects to be discussed are the most important aspects within a prescription talk and what information is needed to find the best drug treatment for that specific patient. When moderating the discussion, a pharmacist follows the previously developed guide for prescription talks (Hauser and Matthes 2017). This guide covers both, important medical aspects of drug treatment and the elements of shared decision making aiming at least at an informed consent. Immediately after the guided peer discussion, which lasts about 10–15 min, the same “doctor” conducts the prescription talk again, but as if it was the first time. This second try is followed by a feedback round giving the student in charge, the simulated patient, the fellow students and the pharmacist tutor the opportunity to share their views on both conversations.

#### Content covered by the second try

In the second encounter, clearly more medical aspects were covered (Table 1). There was an increase with respect to nearly all categories (Table S1). The proportion of “doctors” addressing progress evaluation and obtaining the patient’s consent was more than doubled. While adverse drug effects were only mentioned sporadically in the first encounter, almost all students mentioned this topic after the peer discussion. Of interest, a look at the sub-categories shows that students did not only touch the respective main category more often but gave more detailed information. This is presented exemplarily for the category “adverse drug effects” in Table 2. The only observed decrease referred to the treatment setting (i.e. outpatient or inpatient).

**Table 2:** Frequency of aspects with regard to adverse drug effects mentioned in an impromptu (1^st^ encounter) simulated prescription talk or the simulated prescription talk led by the same student after a short, guided peer discussion (2^nd^ encounter), respectively

Sub-categories of “adverse drug effects”	Frequency during 1^st^ encounters (n = 38 in total)	Frequency during 2^nd^ encounters (n = 38 in total)
Reason	0	10
Vomiting	0	5
Frequency	0	21
Nausea	0	13
Diarrhea	0	36
Rash	1	32
Measures if rash occurs	1	30
Measures if diarrhea occurs	0	21
Diarrhea, when to contact a doctor	0	23

### Learning effects assessed by a written test

**Fig. 1:**
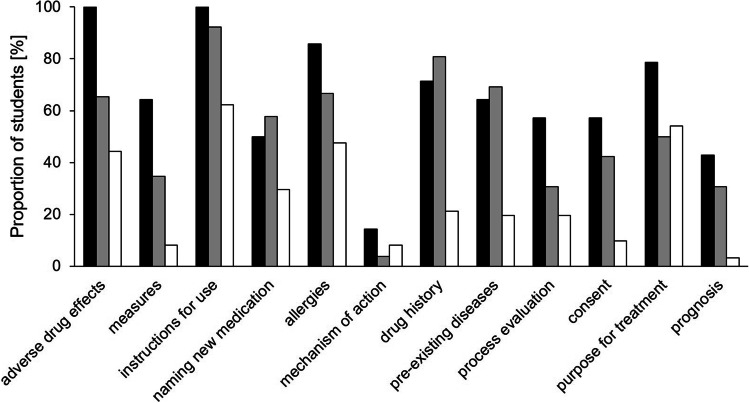
Content of a prescription talk as assessed in a formative written test. 14 students who played the doctor in a simulated prescription talk four days ago (black columns), 26 who watched and discussed this scenario (grey columns), and 61 students attending the same one-week course but not the simulation scenario (white columns) took the test. The proportion of students in each group mentioning an aspect in their description of a prescription talk is given

In two terms, all students who participated in the “PJ-STArT-Block” week were asked to voluntarily take a written test at the end of the course, i.e. four days after the simulated prescription talks. The task was to describe a prescription talk based upon a written case vignette. Comparing test results of students who participated our simulation scenario (i.e. students having acted as a “doctor” or having only observed and discussed the talk) with that of students who have attended the one-week course but not our prescription-talk scenario revealed clear differences with respect to the content of the described conversation (Fig. 1). Students who participated in the prescription-talk scenario clearly outperformed those, who did not participate, in almost all categories (Table S2). Of interest, students who conducted the prescription talk as a “doctor” tended to be even better than the students who only observed and discussed the talks, too. The level of information given about the mechanism of action was constantly low in all subgroups.

## Discussion

We present a pharmacology education module specifically focusing physician–patient communication in a prescription talk. Advanced medical students often feel uncertain in their competencies about a drug therapy (Tobaiqy et al. 2007; Brinkman et al. 2017b; Johannsen et al. 2019). That even graduates often feel insufficiently prepared to cope with the responsibilities of drug prescription suggests that education in clinical pharmacology and therapeutics needs improvement (Han and Maxwell 2006).

### Students’ medical communication in an impromptu prescription talk

As a first important finding, our exploratory study indicates that even advanced medical students may have significant deficits in doctor-patient communication about drug treatment. This indicates that such deficits are not only due to unfavourable working conditions in the daily routine of doctors, e.g. the lack of time.

Simulated prescription talks conducted by medical students shortly before their final practical year mostly lacked essential information like risks of the drug treatment and adverse drug effects. Similar results from our previous study might have been explained by the simulated inpatient setting, i.e. students might have had in mind that in the hospital the patient will be closely monitored and there definitely would be a soon second encounter (Hauser and Matthes 2017). In our current study, an outpatient setting was simulated. Thus, it would have been all the more important to address possible problems with drug therapy and also measures that would then have to be taken to ensure safe treatment. There are several possible explanations for the above-mentioned deficits in students’ prescription talks, e.g. underestimation of the topic’s importance, the intention to not alienate the patient, or simply lack of knowledge. Of note, patients feel information on risks and adverse effects to be particularly important and they are rather dissatisfied with the corresponding information actually provided by their physicians (Barry et al. 2000; Ziegler et al. 2001; Mahler et al. 2009; Twigg et al. 2016). Importantly, several studies could not confirm the concern that informing patients about possible adverse effects of a treatment could have a negative impact on medication adherence, occurrence of side effects or clinical outcomes (Jose and AlHajri 2018). That the observed deficits were due to lack of knowledge is rather unlikely for two reasons. First, 5^th^ year students attending the course usually had successfully passed the two main pharmacology courses, i.e. in basic and clinical pharmacology. Second, in the second try most students conveyed the information lacking before. The discussion between the two meetings was short and condensed, so that the students could at best be reminded of what they had already learned, and there would not have been enough time to convey the relevant aspects to them comprehensively. The rather differentiated presentation of the adverse effects, which can be seen in the number of sub-categories, underlines this interpretation.

In summary, we confirmed the need to improve students’ ability to talk about drug therapy to a patient. Our approach was to support this through immediate repetition of the simulated conversation.

### Differences between first and second encounter

In the second encounter following a brief peer discussion moderated by a pharmacist, conversations became much more comprehensive and informative. Of note, the interim discussion was based upon a conversation guide we previously developed as a tool to improve prescription talks (Hauser and Matthes 2017). As a checklist, the guide has proven to be reliable and discriminative in simulated talks. Recently, we were able to show that the guide can be used in the daily routine of doctors in GP practices and can improve the satisfaction with prescription talks on the part of patients and doctors (Kirsch and Matthes 2021). Our conversation guide considers important drug information as covered by the Medication Communication Index as well as essential elements of shared decision making. This might explain, why students not only gave more information after the guide-based discussion but more frequently obtained patient’s consent. A lack of awareness of the importance of prescribing medicines, partly due to insufficient emphasis on this aspect in medical studies, was repeatedly cited as a cause of corresponding deficiencies and errors (Weingart et al. 2000; Dean et al. 2002; Barber et al. 2003). This might explain both, the deficits during the impromptu conversation and the improvement by the short intervention. In another study we found that an elective on how to lead a prescription talk made medical students feel more confident and aware of the impact of physician–patient communication (Hauser et al. 2017). In that elective, we combine problem-based learning (PbL) and a simulation scenario. It has been shown that PbL particularly fosters performance and skills and thus application of knowledge (Strobel and van Barneveld 2009). In fact, we recently found that medical students’ self-assessment of application-oriented knowledge was lower than of declarative knowledge throughout their studies (Johannsen et al. 2019). A review showed that simulating patient conversations is particularly important in the field of pharmacology education (Aura et al. 2015). It made students generally feeling more confident about identifying, preventing, correcting and communicating medication errors. Furthermore, conducting conversations with simulated patients was described as motivating and increasing the awareness of patient safety.

Taken together, the observed improvement of simulated prescription talks after a brief conversation guide-based discussion was likely due to fostering application of existing knowledge in an application-oriented context.

### Effects indicated by a written test

In a case-based written test four days after the intervention, students who had participated in the simulation module outlined a clearly more complete prescription talk than students who had not participated in this module. Overall students having attended the simulation module thus were able to transfer what they have learned to a similar situation a few days later. Of note, even watching the simulation had a significant effect, although the numerically better performance of the students who played the doctor suggests that active participation may be preferable. Even though we cannot conclude on the sustainability of our intervention, our approach of testing the content of the module a few days later is likely to enhance learning (Roediger and Karpicke 2006). Furthermore, the simulation-based approach should foster active learning and thus retention (Joyner and Young 2006). In a recent clinical study, we showed that a simple intervention (providing the same short conversation guide given to students here) can improve prescription talks (Kirsch and Matthes 2021). Therefore, we are confident that the intervention described here can also have an effect that lasts beyond the course.

### Limitations

The data were obtained with medical students in Cologne. Furthermore, the pharmacology module described here is embedded in a compulsory one-week course preparing 5^th^ year medical students for their final practical year. We thus can neither be sure that our findings can be transferred to other locations nor that our approach would show similar effects if applied isolated, e.g. as an elective. The number of conversations analysed is limited, as of the 56 ‘active’ students available, 18 declined to have their simulated talk recorded. Nonetheless, we consider our data to be meaningful because we conducted a pre-post comparison of the content addressed. However, larger subsequent studies are needed to generalise our exploratory data. In our simulated prescribing scenario, we only focused on prescribing an antibiotic drug treatment. Prescribing deficits in this field seem to be particularly large among final-year medical students (Brinkman et al. 2018). We, in a previous study, also found that students are quite sceptical about this issue: antibiotic drugs have been the only drug class called by name when students were asked about their perceived pharmacological deficits (Kirsch et al. 2019). Although addressing communication of doctors with patients we use pharmacists as supervisors. We do not think that this lowers the learning effects since we emphasise the content of the prescription talk. Of note, there have been repeated calls for pharmacists to be involved in medical education (Brinkman et al. 2017a; Lerchenfeldt and Hall 2018). We cannot conclude on the sustainability of our approach and it is quite likely that especially an approach that aims at a competence that touches not only cognitive but also affective aspects will have to be applied again and again during medical studies. Further studies should aim at putative long-term effects and include further populations, e.g. less and more advanced students and postgraduates.

## Conclusions

We describe a simulation-based module for pharmacology training of medical students. On the one hand, we thus can define students’ deficits with leading a prescription talk. On the other hand, our approach appears to enhance students’ performance during as well as a few days after the module. The international literature suggests that deficits in medical communication occur worldwide (Brinkman et al. 2018). Thus, we assume that elsewhere medical students have the same needs making it likely that these students might benefit from similar approaches. Our approach thus may be an example for training medical students in simulated and clinical environments like the EACPT recommended to improve pharmacology education (Brinkman et al. 2017b).

## Supplementary Information

Below is the link to the electronic supplementary material.

Supplementary file1 (DOCX 20 KB)

Supplementary file2 (DOCX 14 KB)

## Data Availability

As supplemental material and upon request.
