# Supplementary material for: A simulation-based module in pharmacology education reveals and addresses medical students’ deficits in leading prescription talks
Source: Naunyn Schmiedebergs Arch Pharmacol. 2021 Sep 15;394(11):2333–41. doi: 10.1007/s00210-021-02151-w (PMC8514349; doi:10.1007/s00210-021-02151-w)
Supplement: Supplementary file 1 — Supplementary file1 (DOCX 20 KB) [file 210_2021_2151_MOESM1_ESM.docx]

**Table S1:** Frequency of aspects mentioned in an impromptu (1^st^ encounter) simulated prescription talk or the simulated prescription talk led by the same student after a short, guided peer discussion (2^nd^ encounter), respectively.

| Main Category | Frequency during 1^st^ encounters (n=38 in total) | Frequency during 2^nd^ encounters (n=38 in total) | Comparison* of frequencies |
| --- | --- | --- | --- |
| Adverse drug effects | 2 | 37 | <0.001 |
| Mechanism of action | 4 | 8 | >0.05 |
| Prognosis | 9 | 32 | <0.001 |
| Consent | 14 | 29 | 0.001 |
| Progress evaluation | 15 | 31 | <0.001 |
| Allergies | 24 | 36 | <0.01 |
| Drug history | 25 | 37 | <0.001 |
| Setting | 30 | 25 | >0.05 |
| Instruction for use | 32 | 38 | 0.025 |
| Pre-existing diseases | 34 | 35 | >0.05 |
| Purpose of treatment | 38 | 38 | >0.05 |
| Naming new medication | 38 | 38 | >0.05 |

* p-value in a Fisher’s exact test
