# Supplementary material for: A simulation-based module in pharmacology education reveals and addresses medical students’ deficits in leading prescription talks
Source: Naunyn Schmiedebergs Arch Pharmacol. 2021 Sep 15;394(11):2333–41. doi: 10.1007/s00210-021-02151-w (PMC8514349; doi:10.1007/s00210-021-02151-w)
Supplement: Supplementary file 2 — Supplementary file2 (DOCX 14 KB) [file 210_2021_2151_MOESM2_ESM.docx]

**Table S2:** Content of a prescription talk as assessed in a formative written test. Fourteen students who played the doctor in a simulated prescription talk four days ago (SimDoc), 26 who watched and discussed this scenario (WatchDoc), and 61 who attended the same one-week course but not the simulation scenario (None) took the test. Frequency of mentioning an aspect is indicated (percentage in brackets).

| **Content (category)** | **SimDoc** | **WatchDoc** | **None** | **SimDoc**  **vs. None*** | **WatchDoc**  **vs. None*** |
| --- | --- | --- | --- | --- | --- |
| adverse drug effects (ADE) | 14 (100) | 17 (65) | 27 (44) | <0.01 | 0.1 |
| measures [to be taken if ADE] | 9 (64) | 9 (35) | 5 (8) | <0.01 | <0.01 |
| instructions for use | 14 (100) | 24 (92) | 38 (62) | <0.01 | <0.01 |
| naming new medication | 7 (50) | 15 (58) | 18 (30) | 0.2 | 0.017 |
| allergies | 12 (86) | 18 (69) | 29 (48) | 0.015 | 0.099 |
| mechanism of action | 2 (14) | 1 (4) | 5 (8) | 0.61 | 0.66 |
| drug history | 10 (71) | 21 (81) | 13 (21) | <0.01 | <0.01 |
| pre-existing diseases | 9 (64) | 18 (69) | 12 (20) | <0.01 | <0.01 |
| process evaluation | 8 (57) | 8 (31) | 12 (20) | <0.01 | 0.28 |
| consent | 8 (57) | 11 (42) | 6 (10) | <0.01 | <0.01 |
| purpose of treatment | 11 (79) | 13 (50) | 33 (54) | 0.13 | 0.82 |
| prognosis | 6 (43) | 8 (31) | 2 (3) | <0.01 | <0.01 |

* p-value in a Fisher’s exact test
